# Development and performance analysis of Si-CaP/fine particulate bone powder combined grafts for bone regeneration

**DOI:** 10.1186/s12938-015-0042-4

**Published:** 2015-05-22

**Authors:** Chengli Sun, Ye Tian, Wenxiao Xu, Changlong Zhou, Huanxin Xie, Xintao Wang

**Affiliations:** Department of Orthopaedic Surgery, The Second Harbin City Hospital, Harbin, 150056 China; Department of Orthopaedic Surgery, The Second Affiliated Hospital of Harbin Medical University, 246 Xuefu Street, Nangang District, Harbin, 150086 China

**Keywords:** Si-CaP, Fine particulate bone powder, Bone regeneration

## Abstract

**Background:**

Although autogenous bone grafts as well as several bone graft substitute material have been used for some time, there is high demand for more efficient and less costly bone-substitute materials. Silicon-substituted calcium phosphates (Si-CaP) and fine particulate bone powder (FPBP) preparations have been previously shown to individually possess many of the required features of a bone graft substitute scaffold. However, when applied individually, these two materials fall short of an ideal substitute material. We investigated a new concept of combining Si-CaP with FPBP for improved performance in bone-repair.

**Methods:**

We assessed Si-CaP/FPBP combined grafts in vitro, by measuring changes in pH, weight loss, water absorption and compressive strength over time.

**Results:**

Si-CaP/FPBP combined grafts was found to produce conditions of alkaline pH levels compared to FPBP, and scaffold surface morphology conducive to bone cell adhesion, proliferation, differentiation, tissue growth and transport of nutrients, while maintaining elasticity and mechanical strength and degradation at a rate closer to osteogenesis.

**Conclusion:**

Si-CaP/FPBP combined grafts was found to be superior to any of the two components individually.

## Background

The repair and regeneration of bone is a complex interplay between the ground substance, cells and milieu, but is still not fully comprehended [1]. Although autologous bone grafts have been used successfully for many bone disorders for some time now, it is not always practical and can be challenging in harvesting sufficient graft material and causes trauma to the donor site.

There are alternative materials to autologous bone grafts currently used in various bone repairs that have yielded some success. However, there are many technical problems and the osteogenic effect of such alternatives is still not satisfactory [2, 3]. Natural biological materials such as collagen, chitosan and others have problems such as poor mechanical strength, immunogenicity, non-osteoconductivity or non-osteoinducive, inappropriate degradation time or poor reproducibility [4, 5].

Organic polymers such as poly(lactic acid), poly(glycolic acid), poly(ε-caprolactone) and poly(lactic-co-glycolic acid) copolymers have insufficient mechanical strength and are poorly hydrophilic. Degradation of some organic polymers causes acidic conditions that result in inflammation. These materials also have weak adsorption of cells and more importantly: lack cell surface recognition signals and interaction with the biological tissue. In addition, they are still expensive and have unsatisfactory plasticity [1, 6].

With inorganic materials such as β-tricalcium phosphate, hydroxyapatite, and bioactive glass, the scaffold resorption rate exceeds the bone formation rate and cannot maintain repair function. Others absorb too slowly or not at all, can produce foreign body reactions after implantation, are not conducive to new bone remodeling and their production method cannot guarantee consistent pore connectivity [1, 7]. Therefore, alternatives to autogenous bone graft material to accelerate the repair of bone defects has been an important issue faced by orthopedic surgeons.

Calcium phosphate has been commonly used as a substitute scaffold in bone tissue engineering, but easily becomes brittle, has poor degradation performance and absorbs poorly, affecting the formation of new bone and later development [8, 9]. However, silicon-substituted calcium phosphate (Si-CaP) has been found to have good biocompatibility, biodegradability and osteoconduction [10, 11]. The addition of silicone to calcium phosphates causes changes in the structural properties of the scaffolding material and significantly improves bioactivity. These materials become fully absorbed by bone cells and are replaced by natural bone during bone remodeling [12, 13]. Several studies [14–20] have shown that, compared with a pure calcium phosphate, Si-CaPs have the following advantages: (1) good degradation performance, (2) higher biological activity, (3) supports the migration of bone cells and their differentiation and (4) can reduce cell damage caused by environmental changes. Si-CaP is a new scaffold with good prospects. However, although Si-CaP is osteoconducive, it is not osteoinducive [21], which is a major shortcoming of autogenous bone graft substitute materials. Creating a scaffold that is combined with a substance that is involved in osteoinduction may increase the osteogenic potential of the graft substitute material. Studies have shown that fine particles of bone accelerate healing compared to traditional bone grafts [22]. However, since such particles of lose bone structure easily drain, are difficult to mold and have other shortcomings, the application is limited. Therefore, we focused on ways to overcome the respective shortcomings of Si-Cap and fine bone particles in bone repair. To do this, we investigated the potential of viable osteoblasts in fine particulate bone powder (FPBP) to provide osteoinductive and osteogenic properties to the osteoconductive Si-CaP in the combination scaffold: Si-CaP/FPBP, as a tentative method for bone-defect repair.

## Methods

### Synthesis and identification of Si-CaP

Three-dimensional porous Si-CaP tubes were prepared using aqueous precipitation method as described previously [23]. The tubes had an inside diameter, outside diameter and length of 0.6, 1 and 1.5 cm, respectively and weighed 1.5 g. The crystal phase composition of Si-CaP was studied with X-ray diffraction, using a D/MAX-Rb X-ray diffractometer (Rigaku, Japan) with Ni-filtered Cuka radiation operated at 30 kV and 10 mA at a scanning speed of 1/min. Si-CaP functional groups were detected by Nicolet710 Far-infrared Fourier transform spectroscopy (Thermal, United States) with a frequency range from 4,000 to 400 cm^−1^ and a resolution of 2 cm^−1^. Si-CaP microstructure and elemental composition was analyzed using a dual beam focused ion beam scanning electron microscopy (FIB-SEM) system (HELIOS NanoLab 600i, FEI, Netherlands) equipped with an energy dispersive X-ray spectroscopy (EDS) unit operated at 20 kV. Liquid displacement methodology was used to measure the porosity of Si-CaP as described previously [24].

### Preparation of fine particulate bone powder

Animal study was approved by the Institutional Animal Care and Use Committee of Harbin Medical University (Protocol: 2012-002). FPBP was prepared from New Zealand white rabbits (6 months old, 2.5 kg). Briefly, iliac crest bone (1 g) was first ground using an electric ball mill drill (BJ2103, Bojin, China). Particles of 300–500 μm were isolated using a sub-sieve sizer and centrifuged at 120 rpm for 5 min (Eppendorf, Germany). Light microscopy (DVM2500, Lecia, Germany) and scanning electron microscopy (SEM) (HELIOS NanoLab 600i, FEI, Netherlands) were used to characterize the general and surface morphology of FPBP particles.

### Construction of Si-CaP/FPBP scaffold

**Figure 1:**
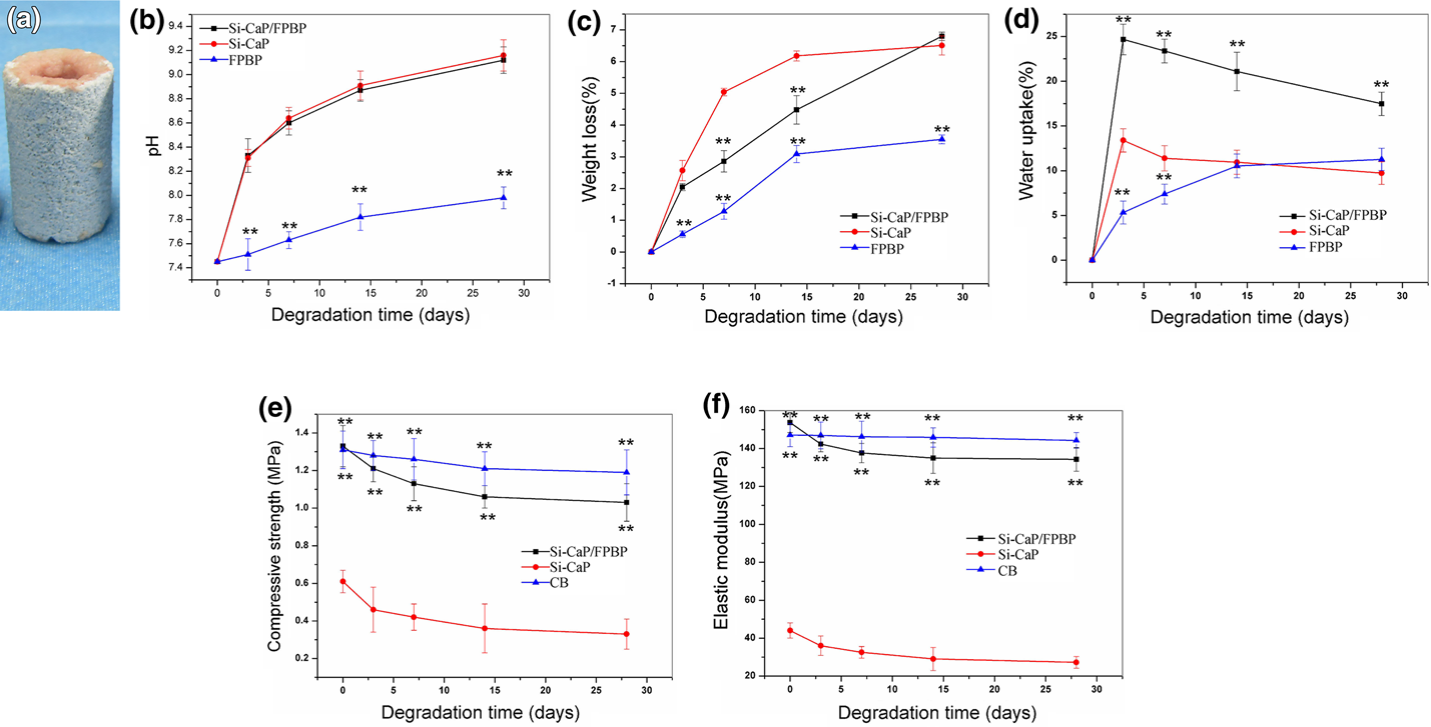
The Si-CaP/FPBP tube (**a**), and the: changes of pH (**b**), weight (**c**), water absorption ability (**d**), compression strength (**e**) and elastic modulus (**f**) after the scaffolds were soaked in DMEM medium for different time periods. n = 7. **p < 0.01 compared to Si-CaP group.

To prepare Si-CaP/FPBP scaffolds, Si-CaP and FPBP were mixed at a 1:1 weight ratio and packed into 1.6 cm^3^ tubes (diameter: 0.92 cm, height: 2 cm). An example of the scaffold is shown in Figure 1a. Si-CaP, FPBP and Si-CaP/FPBP scaffolds were then subjected to in vitro degradation tests at 37°C. Experiments were divided into three groups: Si-CaP group, FPBP and Si-CaP/FPBP. Si-CaP tubes were filled with FPBP at a weight ratio of 1:1 (0.75 g of each component). Bone scaffolds were cultured in six well culture plates containing 15 ml DMEM culture medium (pH 7.4) (Invitrogen, USA). Bone scaffolds were incubated at 37°C for 3, 7, 14 and 28 days.

### pH measurement

The pH change of the culture medium was detected with pH meter at each at 0, 3, 7, 14 and 28 days of incubation. Each average pH value was obtained from seven measurements taken under the same testing conditions. Experiments were performed in seven replicates.

### Weight loss

To measure the weight changes, scaffolds were removed from the culture plates at 0, 3, 7, 14 and 28 days of incubation, rinsed with water and dried for 24 h with a blow dryer. The weight loss values were measured using an electronic balance with an accuracy of 0.001 g. The weight loss ratio was calculated from the equation:

$${\text{weight loss ratio }}(\%) = \left( {{\text{M}}_{0} - {\text{M}}_{1} } \right)/{\text{M}}_{0} \times 100,$$

where M_0_ is the weight of scaffolds before degradation and M_1_ is the weight of dried scaffolds after degredation. Each average weight loss value was obtained from seven measurements taken under the same testing conditions. Experiments were performed in seven replicates.

### Water absorption

Water absorption was measured at 0, 3, 7, 14 and 28 days of incubation. To measure the water absorption, scaffolds were removed from the culture plates and blotted dry on filter paper to remove excess water. The water absorption was measured using an electronic balance with an accuracy of 0.001 g. The water absorption ratio was calculated from the equation:

$${\text{water absorption ratio }}(\%) = \left( {{\text{M}}_{2} - {\text{M}}_{0} } \right)/{\text{M}}_{0} \times 100,$$

where M_2_ is the weight of scaffolds with excess water removed. Each average water absorption value was obtained from seven measurements taken under the same testing conditions. Experiments were performed in seven replicates.

### Compressive strength measurement

The compressive strengths of the scaffolds were determined using Electronic Universal Testing Machine (Instron 5500R, United States) with a crosshead speed of 1.0 mm/min. To conduct the test, the scaffolds were removed from the culture plates, rinsed with deionized water and dried thoroughly using a blow dryer. The load was recorded as compressive strength (MPa) at the point where the scaffold fractured. The elastic modulus (MPa) was determined by the slope of the initial linear portion of the stress–strain curve. Each average value was obtained from seven measurements taken under the same testing conditions. Experiments were performed in seven replicates.

### Surface morphology

The surface morphology of scaffolds were observed using FIB-SEM. Briefly, the scaffolds were removed from the culture plates, fixed with 2.5% glutaraldehyde solution (pH 7.4) (Sigma Chemical Co., USA) for 24 h at 4°C and then for 30 min in 1% osmium tetroxide (Sigma Chemical Co.). The scaffolds were dehydrated in a graded series of ethanol, critically point dried with CO_2_ and sputtered with gold.

### H&E staining

For H&E staining, nuclei was stained with alum haemtoxylin for 10 min and differentiate with 0.3% acid alcohol for 2 s. Then, samples were rinsed in Scott’s tap water substitute and stained with eosin for 2 min. Finally, slides were dehydrated and mounted with permount medium.

### Statistical analysis

All values were expressed as mean ± standard deviation. Differences among groups were analyzed by ANOVA followed by Bonferroni post hoc analyses as appropriate, using SPSS 17.0 statistical software. A p value of <0.05 was considered significant.

## Results

### Synthesis and identification of Si-CaP

**Figure 2:**
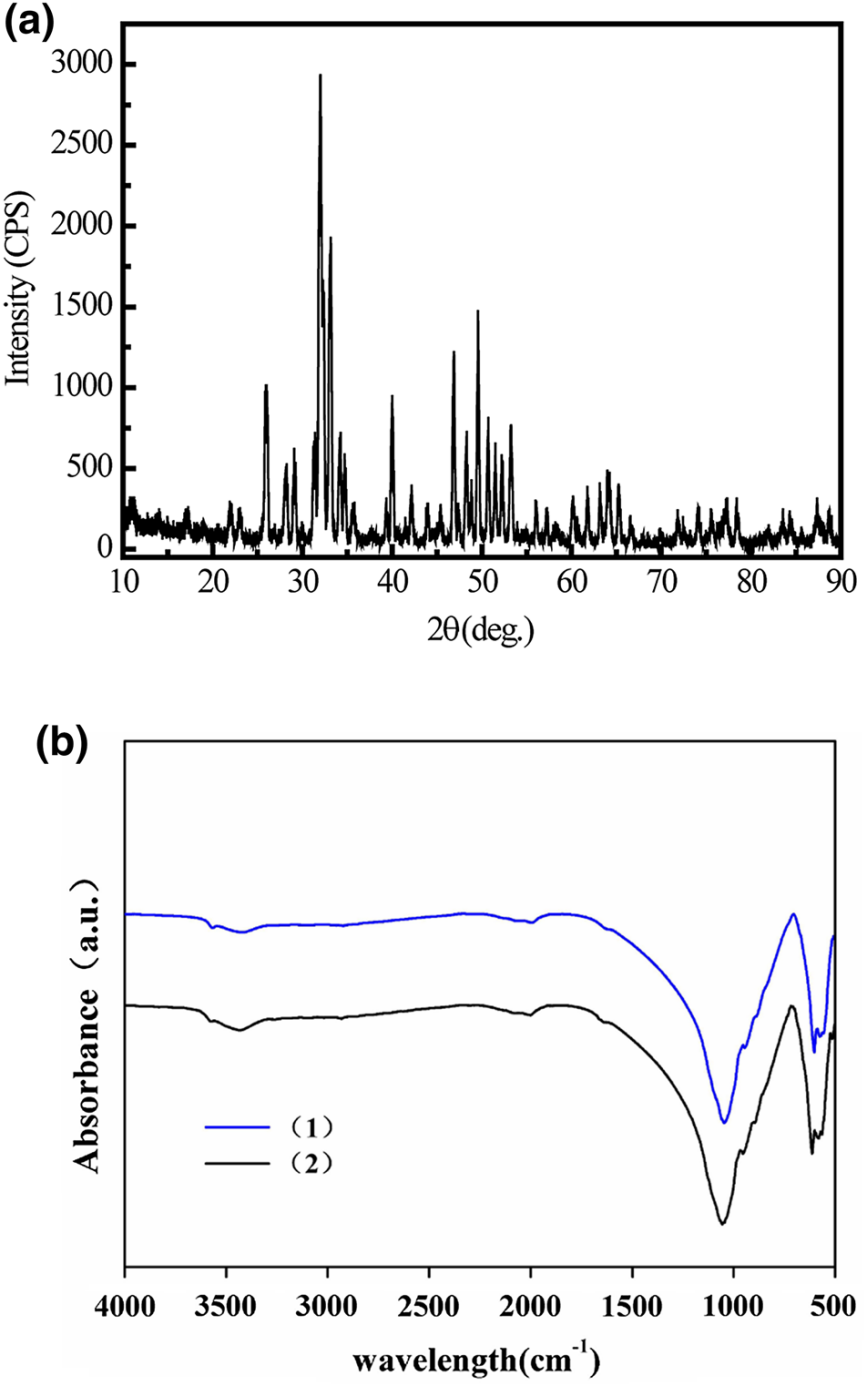
**a** X-ray diffraction spectrum of Si-CaP powder. **b** Far-infrared Fourier transform spectroscopy analysis of two batches (1, 2) of Si-CaP powder sintered at 1,200°C for 4 h.

**Figure 3:**
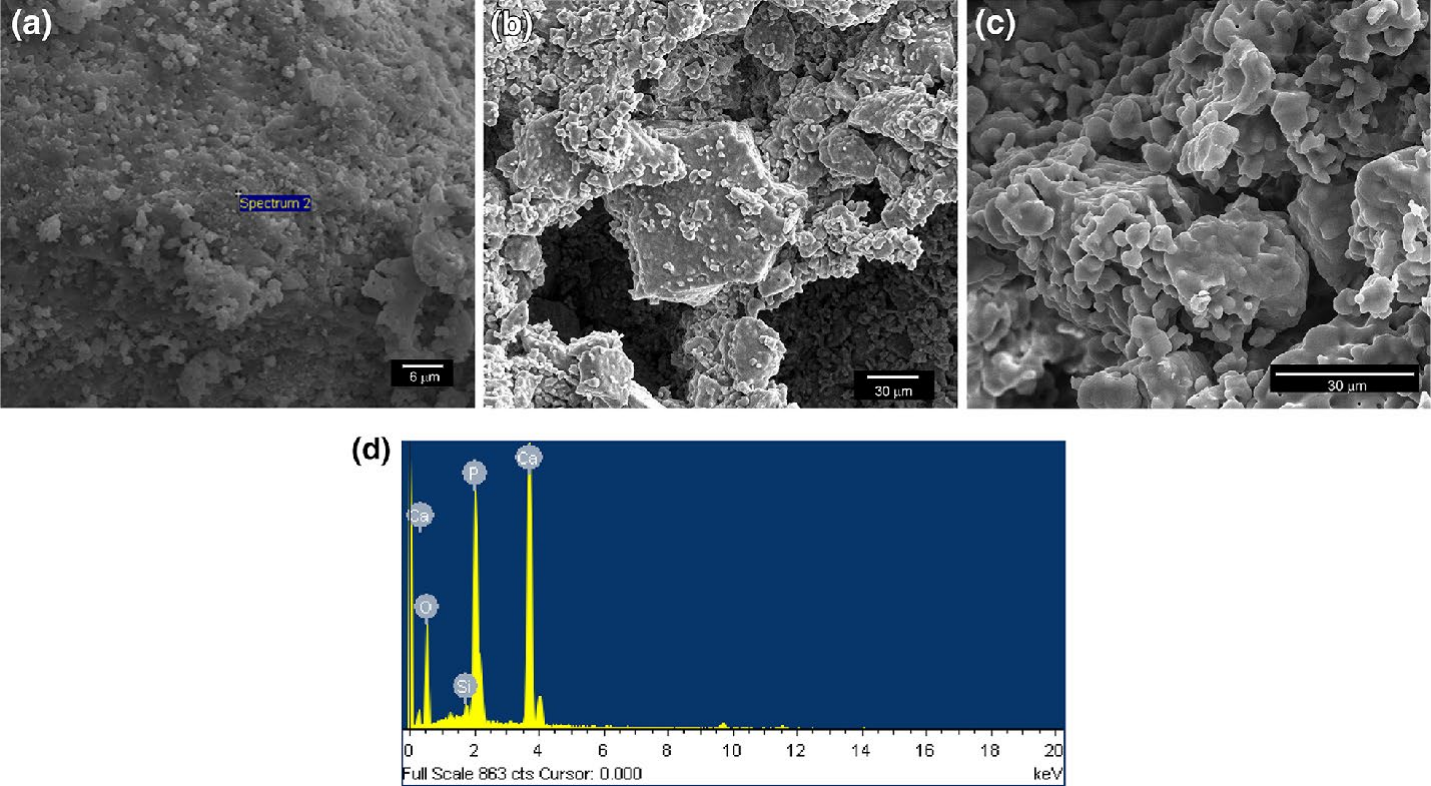
SEM images of Si-CaP powder at 200× (**a**), 1,000× (**b**) and 10,000× (**c**) magnification. **d** EDS profile of Si-CaP powder surface at the spot indicated in **a**.

X-ray diffraction analysis showed that synthesized Si-CaP was homogenous and single phase material with all peaks matching with standard Si-CaP diffraction peaks provided by the International Centre for Diffraction Data (Figure 2a). Far-infrared Fourier transform spectroscopy analysis of two batches of Si-CaP exhibited same absorbance spectrum with major bands at 1,125 and 692 cm^−1^, demonstrating the repeatability of this synthesis method (Figure 2b). The images obtained from FIB-SEM showed rod or spherical shapes of Si-CaP particles with small Si-CaP crystals clustered on the surface (Figure 3a–c). Nanoscale pores with irregular sizes and shapes, formed by Si-CaP crystal clusters, were present on particle surfaces. Macropores of 150–300 µm were evenly distributed and connected by micropores of 1–10 µm throughout the scaffolds. EDS point analysis detected 8% silicon in the Si-CaP (Figure 3d; Table 1). Additionally, as determined by liquid displacement methodology, the average porosity of Si-CaP was 74.144 ± 3.833%.

**Table 1:** Elemental analysis of Si-CaP by EDS

Element	Weight (%)
O K	50.09
Si K	0.8
P K	16.48
Ca K	32.63
Total	100.00

### Preparation and characterization of FPBP

**Figure 4:**
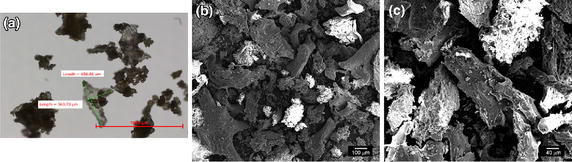
**a** Light microscope image of FPBP. FPBP particle sizes were about 300–500 µm. **b**, **c** SEM images of FPBP at 200× (**b**) and 1,000× (**c**).

Bright field images showed that FPBP particles had irregular shape with bone debris scattered on the surface (Figure 4a). SEM was used to characterize the surface morphology of FPBP (Figure 4b, c). The FPBP was observed as a mixture of 10 μm porous cortical and cancellous bone. The majority was coarse cancellous bone.

### Preparation and characterization of Si-CaP/FPBP scaffold

An example of the obtained Si-CaP/FPBP tubes is shown in Figure 1a.

#### pH change

The pH of DMEM solution during the in vitro degradation of three types of scaffolds was first determined. As degradation time increased, the pH of the DMEM solution in all three groups increased. The pH values of Si-CaP degradation solution were slightly higher than that of Si-CaP/FPBP degradation solution, but with no significant difference (P > 0.05). However, the pH values of both Si-CaP and Si-CaP/FPBP degradation solutions were significantly higher than the pH value of the FPBP solution (Figure 1b, P < 0.05).

#### Weight loss

As shown in Figure 1c, Si-CaP and Si-CaP/FPBP scaffolds experienced dramatic weight loss during the 28 days of incubation. Si-CaP scaffolds degraded significantly faster than Si-CaP/FPBP composite scaffolds between days 3 and 14. In contrast, the FPBP group experienced significantly slower weight loss than both Si-CaP and Si-CaP/FPBP groups (P < 0.05).

#### Changes in water absorption

The water absorption of Si-CaP and Si-CaP/FPBP scaffolds can be divided into two phases during the in vitro degradation process: 0–3 days, water absorption increased significantly, and 3–28 days, water absorption appeared to decrease slowly. In contrast, water absorption by FPBP increased slowly, and after 14 days apparent water absorption did not change significantly. Overall, scaffolds in Si-CaP/FPBP group had the highest water uptake amongst the three groups (Figure 1d). However, water absorption was assessed by differences in weight and after 3 days, change in weight is likely due to scaffold-dissolution, rather than loss of absorbed water.

#### Compressive strength

The change in compressive strength of Si-CaP, Si-CaP/FPBP and cortical bone (CB) was measured and compared (Figure 1e). The strength of scaffolds in each group continuously decreased during the 28 day period. Before in vitro degradation, the average compression strength of Si-CaP/FPBP, Si-CaP and CB was 1.288 ± 0.107, 0.614 ± 0.130 and 1.200 ± 0.130 MPa, respectively. Before degradation, the compression strength of Si-CaP/FPBP scaffolds was significantly higher than that of Si-CaP scaffolds, but lower than that of CB blocks (P < 0.05).

#### Elasticity modulus

Before in vitro degradation, the elasticity modulus of Si-CaP/FPBP, Si-CaP and CB blocks were 149.705 ± 7.414, 43.033 ± 3.933 and 143.976 ± 4.630 MPa, respectively. The elasticity modulus of scaffolds in Si-CaP, Si-CaP/FPBP groups continuously decreased over the 28 days. As shown in Figure 1f, the elasticity modulus of Si-CaP was significantly lower than the Si-CaP/FPBP. The elasticity modulus of Si-CaP/FPBP scaffolds was slightly lower than CB blocks, but not significantly different (P > 0.05).

#### SEM observation of scaffolds

**Figure 5:**
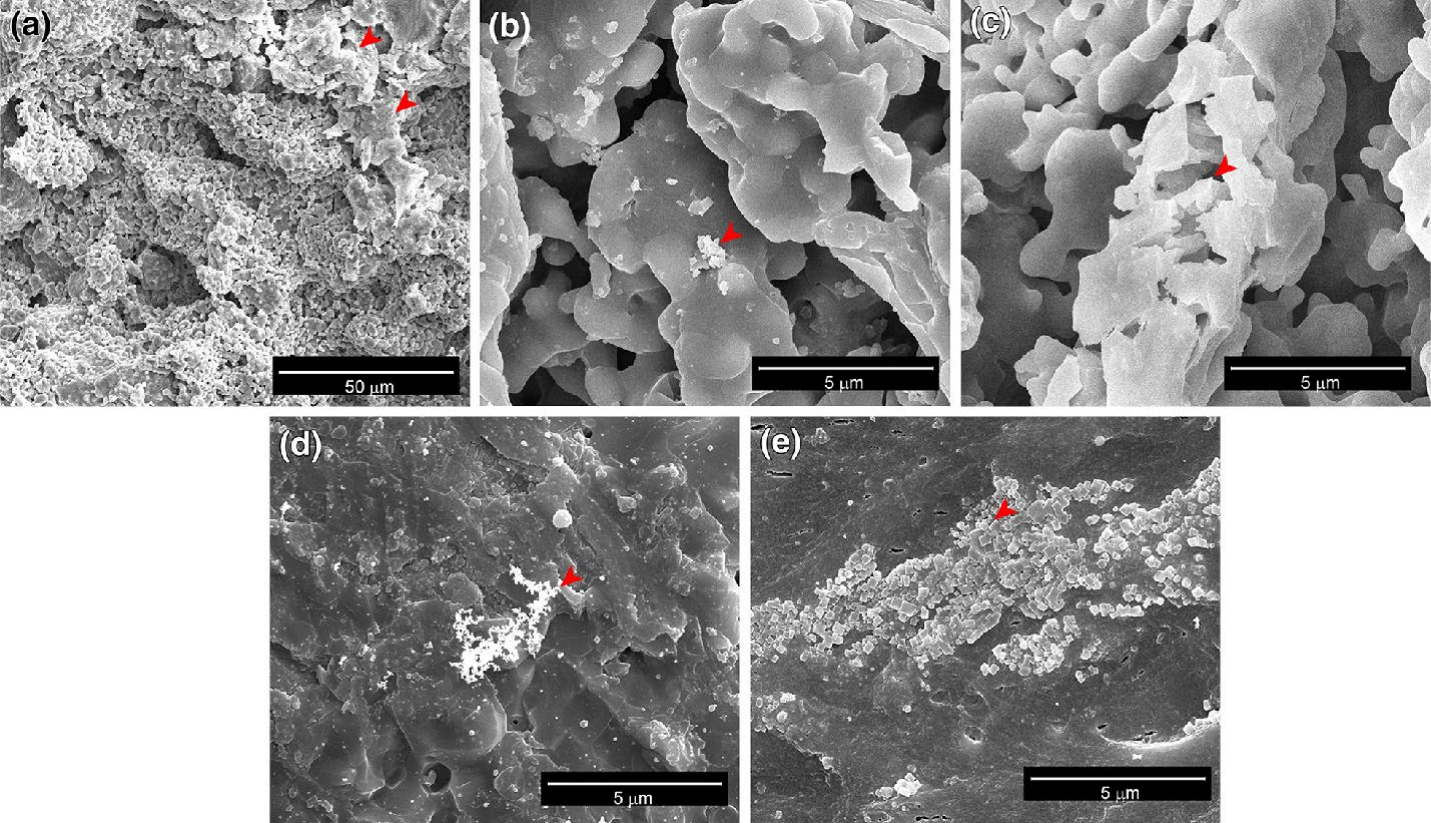
SEM images of Si-CaP/FPBP at day 0 (**a**), 3 (**b**), 7 (**c**), 14 (**d**), and 28 (**e**) days in vitro degradation. n = 7.

Cross-section surface morphology of Si-CaP/FPBP scaffold was observed with SEM before in vitro degradation. We found that FPBP attached tightly to the surface of Si-CaP particles (Figure 5a). Si-CaP particles showed uniform short rod shape with size of approximately 2–10 µm. Si-CaP particles and FPBP were relatively uniform and positioned closely together. The Si-CaP/FPBP scaffold formed three-dimensional porous structures. The sizes of pores were approximately 150–300 µm with micropores of 1–10 µm distributed on the relatively smooth pore wall.

After 3 days degradation in DMEM medium, we found Si-CaP/FPBP scaffolds partially degraded and single small spherical particles deposited on the scaffold surface (Figure 5b). Each spherical particle was composed of several apatite crystals, observed by high-powered microscopy (data not shown). The Si-CaP and FPBP scaffolds showed no change (data not shown). At 7 days of in vitro incubation, we found the cell wall of Si-CaP/FPBP scaffold was degraded. Lamellar bone-like apatite had formed on the scaffold surface and a large number of small bone-like apatite particles were deposited on the pore wall (Figure 5c). At the same time point, Si-CaP and FPBP scaffolds surface was only slightly degraded. At 14 days of in vitro incubation, a large number of loose, spherical or short rod-like bone-like apatite particles had deposited on the surface of Si-CaP/FPBP scaffolds (Figure 5d). Finally, at 28 days, a large number of regular lamellar bone-like apatite particles with size approximately 0.2–1 µm were observed on the Si-CaP/FPBP surface (Figure 5e).

#### H&E observation

**Figure 6:**
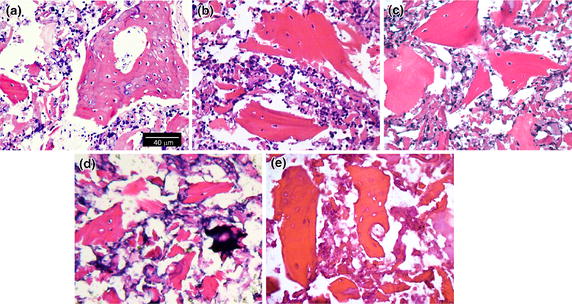
H&E images of Si-CaP/FPBP at day 0 (**a**), 3 (**b**), 7 (**c**), 14 (**d**), and 28 (**e**) days in vitro degradation. n = 7.

H&E observation found that Si-CaP and FPBP engaged tightly with each other at the interface on day 0. As time increased, Si-CaP at the junction between Si-CaP and FPBP became sparse and loose. Lacunae gradually became empty. After 14 days of in vitro incubation, osteoblasts had lyzed and disappeared (Figure 6a–e).

## Discussion

Calcium phosphates are the most frequently used materials in bone substitutions today, due to their similarity to natural inorganic compounds in bone [25–27]. Patel et al. [28] showed that incorporation of silicate ions into hydroxyapatite significantly improves bioactivity. Here, we show that the combination of fine bone particles with silicone-calcium phosphate, further improves the performance of the scaffold.

### Degradation and mechanical properties of Si-CaP/FPBP in vitro

Tuck et al. [25] performed a degradation experiment with Si-CaP in deionized water at a 1:30 mass to volume ratio. After 30 days, the pH of the water had increased from 7.4 to approximately 9 and stabilized there. Analysis by multiple methods showed that Si-CaP had good degradation performance and formed bone-like apatite in vitro. Similarly, the pH of the DMEM for the Si-CaP/FPBP group and the Si-CaP group in our experiment also increased significantly over time and reached approximately 9 after 28 days. FPBP alone only had a modest increase in pH. This change is mainly due to the interaction of oxygen in the phosphate group of the Si-CaP surface with water, producing hydroxide. The reaction is as follows: PO_4_^3−^ + H_2_O = HPO_4_^2−^ + OH^−^ [25]. Further to this, the dissolution of Si-CaP releases Ca^2+^ into the DMEM culture medium as the charge-compensating Si-CaP absorbs two H^+^ ions, resulting in the pH of the DMEM culture increasing further [25]. The pH of Si-CaP/FPBP group was lower than the pH of Si-CaP group throughout the entire degradation process. This may be due to the interaction of Si-CaP with FPBP making the overall degradation rate relatively low and closer to osteogenesis. The surface of Si-CaP/FPBP scaffold degraded completely after 7 days and increasingly formed apatite deposition over the 28 days. The surface of FPBP and Si-CaP scaffolds had degraded slightly at 7 days of incubation with the deposition of some bone-like apatite particles, but there was no significant change after 28 days. This may be due the hydroxyapatite slowly dissolving and escaping from the FPBP. The ability of a scaffold to bond to the living material that surrounds it, as well as maintaining non-acid conditions, is essential to the healing process. The cross-linking of collagen chains during bone formation requires alkaline conditions [29]. Formation of bone-like apatite on the surface of a bone-substitute scaffold is crucial to bone-bonding and facilitates osteoblast adhesion and proliferation. In vitro evaluation of a material’s apatite-forming ability also correlates well with its bone-bonding ability in vivo. Therefore, making in vitro observations of apatite formation is a useful way of preliminary screening of new scaffolding materials before in vivo assessment [28, 30, 31].

The weight loss of the Si-CaP, FPBP and Si-CaP/FPBP groups increased continually as incubation time increased, indicating that the three groups of materials degraded continually in DMEM. At 0–3 days of incubation, the degradation of the amorphous region of the amorphous phase in the Si-CaP group and Si-CaP/FPBP group resulted in the release of large amounts of degradation products, such as PO_4_^3−^ and Ca^2+^, and caused weight loss to increase rapidly in the Si-CaP and Si-CaP/FPBP group. However, after 3 days incubation, plasma PO_4_^3−^ and Ca^2+^ reached heterogeneous nucleation sites and formed bone-like apatite depositions, resulting in decreased weight loss in the Si-CaP and Si-CaP/FPBP group [32]. In the period between 3 and 14 days, the FPBP of Si-CaP/FPBP group degraded and released hydroxyapatite particles that deposited on the surface of the scaffold reducing the weight loss in the Si-CaP/FPBP group compared to the Si-CaP group. The lower rate of weight loss of the Si-CaP/FPBP may be due to dense tissue structure, the presence of hydrophobic material and highly crystalline inorganic components.

Water absorption in the Si-CaP, FPBP and Si-CaP/FPBP groups increased over the first 3 days. This is probably due to a large amount of hydroxyl within the surface and internal pore wall of these porous structures which attracts water molecules through the process of hygroscopy [25]. Additionally, the Si-CaP group, FPBP group and Si-CaP/FPBP group had multiple micropores connected to each other, forming a capillary network structure which may absorb water by capillary action [33, 34].

The ideal autologous bone substitution materials must have good mechanical performance and changes in mechanical strength of autologous bone substitute materials should match the growth rate of bone tissue [35]. In our experiment the compressive strength and elastic modulus of the Si-CaP/FPBP group and cancellous bone group was significantly higher than that of the Si-CaP group and throughout the in vitro degradation process, there was no significant difference in compressive strength and elastic modulus between Si-CaP/FPBP group and cancellous bone group. This indicates that the addition of fine bone particles can make homogeneous composite scaffolds significantly more stress resistant. The elastic modulus of the composites was also improved by the addition of FPBP, which is advantageous in surgical applications. In addition, we observed by SEM that Si-CaP and FPBP distributed uniformly and combined to a well-knit interface. This combination and distribution at the interface provides a structural basis for good mechanical properties of Si-CaP/FPBP. Our results show that FPBP affects the degradation rate of the Si-CaP and improves its mechanical strength. Throughout the degradation process, the change in pH, weight loss, water absorption and mechanical strength indicates that Si-CaP/FPBP had better degradation and mechanical properties than Si-CaP alone.

### Morphology and structure of Si-CaP/FPBP

The microstructures of porous scaffolds have been shown previously to play a vital role in new bone growth, and a pore size of at least 100 µm is a requirement for complete biological activity [36, 37]. One study reported that a scaffold exhibited osteoconductive properties when the pore size was larger than 200 μm [38]. Kang et al. [39] showed that a scaffold with 57% of porosity and pore size of 100–250 μm had good in vitro degradation performance and formed bone-like apatite. Zhuang et al. [40] found that scaffolds with 36–55% porosity and pore size of 200–400 μm had good mechanical properties and a controlled degradation rate.

The Si-CaP/FPBP scaffold constructed in our experiment had a three-dimensional porous structure with pore size about 150–300 μm (“macropores”) and porosity of 75%. Macropores guide the growth of cells and blood vessel on the wall of the pore, which increases osteoconductive performance of the scaffold and bone tissue growth into the internal scaffold [41, 42]. In addition to macropores, micropores of approximately 1–10 μm were observed in the pore walls, by SEM. These micropores were channels that interconnected the larger pores of the Si-CaP/FPBP with good connectivity. Micropores have also been previously found to play important roles in osteogenesis. Yuan et al. [37] found that calcium phosphate scaffolds lacking micropores do not exhibit bone in-growth in dogs, and it is believed that micropores facilitate protein interaction, cell attachment, cellular development and orientation and directionality of cellular growth [43, 44]. Although micropores are not the osteogenic agent itself, their presence results in larger surface area which probably results in more protein absorption (for example BMP), and also ion exchange and apatite formation by dissolution and reprecipitation [37]. BMP is known to induce bone-formation in a dose-dependent manner with a local concentration threshold and a rough surface facilitates attachment, proliferation and differentiation of bone forming cells [37].

During the in vitro degradation process, porosity of Si-CaP/FPBP increased, the pore wall degraded, micropores formed and connectivity between pores increased. A small amount of bone-like apatite formation was observed on the surface of Si-CaP at the seventh day of degradation in DMEM culture medium. This was consistent with the reported literature in which low crystallization apatite was observed on Si-CaP on the seventh day of degradation in simulated body fluid [28]. Compared with the Si-CaP, Si-CaP/FPBP had a more rapid formation of bone-like apatite. The formation of apatite is a natural event in bone formation and important for bioactivity and differences in apatite formation rate between different Si-CaP scaffolds affects bioactivity [45].

### Biological properties of Si-CaP/FPBP

In our experiment, the complete structure of osteoblasts in Si-CaP/FPBP was observed by H&E staining. After in vitro degradation for 14 days, the osteoblasts had dissolved and disappeared. In vivo studies have also shown that FPBP participates in all stages of bone defect repair,plays roles in osteogenesis [46]. Thus the addition of FPBP to Si-CaP should improve the biological activity of the scaffold and our findings support this idea.

Our data taken together and with the support of other works, show that a scaffold comprised of a porous Si-CaP structure packed with fine particles of bone provides a mileu conducive to osteogenesis, bone-bonding and bone repair, whilst maintaining mechanical strength and elastic modus. Although we only performed in vitro experiments in our studies, others have shown that such assessments correlate well with in vivo observations. Hence we expect the Si-CaP/FPBP scaffold will also perform well in vivo.

## Conclusions

Si-CaP and FPBP have both been shown previously to have properties that can facilitate bone-repair. However, individually these materials are insufficient for sustaining bone remodeling. Scaffolds constructed from a combination of the two, on the other hand, may collectively possess the required characteristics for efficient bone-repair. In this study, we found that FPBP enhances the performance of a silicate-substituted calcium-phosphate scaffold. We believe that Si-CaP/FPBP scaffolds may possess the necessary characteristics required of an autologous bone graft substitute for successful bone-repair.

## Abbreviations

CB: cortical bone
FIB-SEM: focused ion beam scanning electron microscopy
FPBP: fine particulate bone powder
Si-CaP: silicon-substituted calcium phosphate.
